# Supplementary material for: Learning curve and surgical outcome of robotic assisted colorectal surgery with ERAS program
Source: Sci Rep. 2022 Nov 29;12:20566. doi: 10.1038/s41598-022-24665-w (PMC9709162; doi:10.1038/s41598-022-24665-w)
Supplement: Supplementary file 1 — Supplementary Information 1. [file 41598_2022_24665_MOESM1_ESM.zip › LSC Raw data-Ñ[▒K/079/H_ñw╝╨░O▒Kñσ.pdf]

正式摘要(電子簽章：000000190725757) 編輯	W85 / W85	列印	英文	【讀取】憑 證
標題(注意:無英文姓名-請補病人基本檔!) 個人範本 離開				

[redacted]

(9)入院日期	109/04/27 (2020) 直腸外科 W85 - 036
(10)轉科日期	
(11)出院日期	109/05/14 (2020) 住院天數計 17 日

診斷

- 入院 :1. Colon Cancer, with liver metastasis, c T4 Nx M1a, Stage IVA, s/p Folfiri and Vectibix x12
  2. Acute right hemiparesis, suspected recurrent stroke, suspected C-myelopathy, under regular medicine
  3. Type 2 DM
  4. HCVD
  5. Dyslipidemia (hypertriglyceridemia).
- 出院 :1. Colon Cancer, with liver metastasis, cT4NxM1a, Stage IVA, s/p Folfiri and Vectibix x12 ;
  s/p laparoscopic AR and conventional multiple liver metastasectomy with cholecystecomy on 2020/05/01, pT3N0M1a
  # With favored reactive RLL pleural effusion (CXR: 2020/05/13)
  2. Acute right hemiparesis, suspected recurrent stroke, suspected C-myelopathy, under regular medicine
  3. Type 2 DM
  4. HCVD
  5. Dyslipidemia (hypertriglyceridemia).

主訴

- for scheduled operation

病史

- This 71-year-old male has history of
  1. Colon Cancer, with liver metastasis, c T4 Nx M1a, Stage IVA, s/p Folfiri and Vectibix x12
  2. Acute right hemiparesis, suspected recurrent stroke, suspected C-myelopathy, under regular medicine
  3. Type 2 DM
  4. HCVD
  5. Dyslipidemia (hypertriglyceridemia).
  According to patient, she suffered from abdominal pain initially. LMD sono revealed colon cancer, hence she went to our CRS OPD for help. Under series survey, colon cancer with liver metastasis was diagnosed. 12 times Folfiri with Vectibix was done, and followed CT showed responce. After discussed with family and general surgeon, this time, she was admitted for combined LAR and liver metastasectomy.

體檢發現

OP FOR LIVER METASTASECTOMY WAS ARRANGED

- General appearance :
  - easy looking , conscious clear , without cardiopulmonary distress
  - Vital signs自動帶入date 體溫(BT) 脈搏(P) 呼吸(R) 血壓(BP) 身高(BH) 體重(BW) BMI
    20200427 37.3℃ 105次/分 19次/分 158/94mmHg 154cm 50kg 21.1
  - Vital signs: B.P. 158/94 mmHg , P.R. 105 /min , R.R. 19 /min , B.T. 37.3 ℃
- HEENT :
  - Eyes: no anemic conjunctiva, no icteric sclera, normal EOM
  - ENT: no otorrhea, no rhinorrhea, no erythematous mucosa, no injected tonsil, no deformity
- Neck: supple, freely movable, trachea midline,no deformity, no engorged jugular vein , no palpable thyroid or lymph node enlargement
- Chest:
  - Inspection: Grossly normal, symmetric and fully expansion, no accessory muscle used, no operation scar, no spider angiomata, no gynecomastia
  - Palpation: no palpable mass or nodule, no subcutaneous emphysema, no tenderness
  - Ausculation: clear breathing sound
- Heart:
  - Palpation: no thrill, no heave
  - Percussion: normal heart size
  - Ausculation: regular heart beat, normal S1, S2, no gallop, no murmur
- Extremities: No deformity, full ranged of motion without limitation, no clubbing finger or nail cyanosis, no pitting edema, no ulcer or pigmentation, no obvious skin lesion, no varicose vein, no bone, joint or muscle tenderness

CRS CONDITION

- Karnofsky performance status: (100) Normal, no complaints, no evidence of disease
- PE of Abdomen:
- CEA: 112.5->5.15 ng/ml

- Imaging Studies:
- Colonoscope report: rectum tumor
- Sono , CT of abdomen and chest Films:liver metastasis
- Figures:
  1. colon & rectum

手術日期及方法

手術日期及方法20200501 - Mesohepatectomy (right anterior section with left medial section).
- Subsegment hepatectomies of lfet lateral section and right posterior section.
20200501 1.Low anterior resection with colo-anal anastomosis2.Ileostomy3.Laparoscopy4.

住院治療經過

After admission, we prescribed complete pre-operative survey. Heart function fair. ICG-15'th minute retention rate was 5.06%. But she couldn't cooperate lung function test, hence we consulted CCRT for pre-OP respiratory training. Her conditon was fair without specific complaint. Base on general surgeon's schedule, combined operation was performed on 2020/05/01. She received laparoscopic AR, multiple liver metastasectomy and protective loop-ileostomy on 2020/05/01. Under the concern of prolonged survery with blood loss, she was sent to SICU on 5/01 for post operation care. At SICU, Extubation was smoothly performed on the following day (5/02). Patient also could gradually tolerate oral intake without obvious abdominal discomfort. Hypernatremia was noted, and kept closely monitoring with fluid adjustment. She was transferred back to ordinary ward on 5/06. We kpet encouraging her for more oral intake. The pelvic drainage tube was removed on 5/07. Due to persisted leukocytosis, we arranged abd sono for F/U, and some pleural effusion over RLL was noted, favored reactive related. Since her condition remained relative stable without SOB and could tolerate oral intake smoothly, we discharged her and arranged OPD for F/U. Danger signs were all well informed to the family.

合併症

一般檢查

累積報告 :

DATE	TIME	ALB	TP	BIL.T	BIL.D	ALKP	AST	ALT	LDH	CHOL	TG	HDL-C	LDL	UA	NH3	AMY
1090427	1422	3.9		0.43	0.12	177	13	10	235							
1090430	0914		6.0							140	114	40	82	2.8		
1090502	0003			1.2	0.5											
1090502	0547	3.0		1.20	0.49	106	3394	1868								
1090503	0530			1.65	0.64		476	941								
1090504	0526			1.7	0.7		84	443								
1090504	1610	3.7														
1090506	0547			1.43	0.72	175	21	110								
1090508	0654	3.4		1.29	0.63	245	15	28								
1090511	1001	3.0		1.15	0.47		24	15								
1090513	1619	3.2		0.80				22								

DATE	TIME	WBC	RBC	HGB	HCT	MCV	PLT	BLST	NEUT	BAND	SEG	LYM	MON	EOS	BAS
1090427	1422	6960	4.80	13.8	43.8	91.3	322		67.3			22.7	9.3	0.1	0.6
1090502	0003	11870	3.28	9.9	29.5	89.9	187			2.0	84.0	9.0	4.0	0.0	0.0
1090502	0546	9010	2.94	8.9	26.3		172			2.0	90.0	2.0	4.0	0.0	0.0
1090503	0526			8.7											
1090504	0526	9540	2.83	8.5	25.4	89.8	125		82.6			11.1	6.2	0.0	0.1
1090506	0545	11310		9.5	28.4		151		80.5			8.9	10.5	0.0	0.1
1090508	0653	13530		10.7	33.6		190		84.2			9.6	5.7	0.4	0.1
1090511	1008	15090		11.5	37.5		348				89.6	6.4	3.2	0.5	0.3
1090513	1621	13550	3.41	10.5			399		85.2			8.2	5.9	0.6	0.1

DATE	TIME	NA	K	CL	CA	BUN	CREAT	Mg	P	CRP	ESR	PCT	LIPASE	LACTATE
1090427	1422	138	4.5		9.0	18	0.51							
1090430	0914									0.878				
1090502	0003	155	3.5				0.59							
1090502	0547	153	3.2	118	8.1	11	0.57							
1090503	0530		2.8											
1090504	0526	148	2.6	116	8.0	18	0.43	1.7	1.1					
1090504	1610		3.0											
1090504	2324		3.2											
1090505	0546		3.5					2.0	1.5					
1090506	0547	156	3.6		8.7	31	0.46							
1090506	1412	153	4.5											
1090508	0654	156	4.6		8.3	45	0.63							
1090511	1001	137	5.0		7.6	32	0.68							
1090513	1619	136	4.5	105	8.0	24	0.72							

DATE	TIME	GLUCOSE	PAB	TRF	ZN	CU	KETONE	CK	CKMB	PT-P	PT-C	APTT-P	APTT-C	TRO-T
1090427	1422	307								10.0	10.3	24.8		
1090430	0906	99												
1090506	0545									15.4	10.3	57.7		

DATE	TIME	SP-GR	PH	PROT	GLUC	KETON	BILI	UROB	OB	NITR	LEU	RBC	WBC	EPCEL
1090502	1706	1.028	6.0	Negative	>1000.0	1+	Negative	0.2	Negative	Negative	Negative	0-2	0-2	0-1

DATE	TIME	PH	PCO2	PO2	SO2	BEB	HCO3	TCO2	HGB	FIO2
1090502	0002	7.440	29.0	404.2	100.0	-4.1	19.3	20.2		
1090504	0520	7.501	31.7	68.9	95.3	1.2	24.2	25.2		

放射線報告 :檢驗項目 **:SONO,ABDOMEN** 報告日期 **:1090513**
主　　訴 :

Result:
Finding:
Sonography of the abdomen was performed and showed:
1. Fatty liver
2. S/P hepatectomy and cholecystectomy
3. Multiple hyperechoic and hypoechoic lesions about 13-21 mm in size are noted over right lobe of liver. As compared with previous CT image, it could be compatible with liver metastasis.
4. The size of right kidney is about 86 mm in length. The size of left kidney is about 94 mm in length.
5. Mild ascites
6. Right pleural effusion
7. No significant abnormal findings of the visible pancreas, spleen and bilateral kidneys
8. Otherwise, no significant abnormal findings
Impression:
As above description.

檢驗項目 **:ABDOMEN (KUB),SUPINE** 報告日期 **:1090511**
主　訴：
Result:
Finding:
> Much bowel gas and fecal material are noted.
> Partially obscured margin of bilateral psoas muscle and bilateral kidney shadows due to bowel gas
> Ostomy over RLQ of abdomen
> Generalized osteoporosis
> Degenerative change of thoracic and lumbar spine with marginal spur formation
> On drainage tubes over upper abdomen
> Surgical clips retention over RUQ of abdomen, lower abdomen and pelvic regions
> Surgical autosuture retention over pelvic region
> s/p internal fixation along Rt femur
Impression:
As above description.

檢驗項目 **:CHEST AP VIEW (PORTABLE)** 報告日期 **:1090507**
主　訴：
Result:
Finding:
> Cardiomegaly with bilateral hilum prominence
> Atherosclerotic changes of thoracic aorta.
> Increased hazy opacities and interstitial infiltrates in bilateral lungs
> Bilateral apical pleural thickening and mild blunting CP angle, r/o pleural effusion or chronic pleural change
> DJD change of thoracic and lumbar spine with osteophytes formation.
> S/P port-A catheter implantation, on EKG lead, central line
> Clips retention and s/p drain tubes insertion over upper abdominal region
Impression:
As above description.

-

特殊檢查

病理報告

活體組織切片病理報告　手術日期：**109/05/01**　病理號：**S0912175**

診斷醫師:李欣倪 報告日期:109/05/08
Pathologic diagnosis:
1. Sigmoid colon, anterior resection --- Adenocarcinoma, moderately differentiated, with focal extracellular mucin production. Tumor invaded to mesocolic soft tissue. The proxiaml cut end and mesocolic lymph nodes (0/22) are free of tumor.
2. IMA root, dissection --- Unremarkable (0/3).
3. Colon, cut end, distal, resection --- Unremarkable.
4. Liver, LL1, hepatectomy --- Metastatic adenocarcinoma, moderately differentiated, consistent with colorectal origin. The resection margin is involved.
5. Liver, LL2, hepatectomy --- Metastatic adenocarcinoma, moderately differentiated, consistent with colorectal origin. The resection margin is involved.
6. Liver, LL3, hepatectomy --- Metastatic adenocarcinoma, moderately differentiated, consistent with colorectal origin. The resection margin is very close (1mm).
7. Liver, LL4, hepatectomy --- Metastatic adenocarcinoma, moderately differentiated, consistent with colorectal origin. The resection margin is involved.
8. Liver, LL5, hepatectomy --- Metastatic adenocarcinoma, moderately differentiated, consistent with colorectal origin. The resection margin is involved.
9. Liver, LL6, hepatectomy --- Metastatic adenocarcinoma, moderately differentiated, consistent with colorectal origin. The resection margin is involved.

10. Liver, S6-1, hepatectomy --- Metastatic adenocarcinoma, moderately differentiated, consistent with colorectal origin. The resection margin is involved.
11. Liver, S6-2, hepatectomy --- Metastatic adenocarcinoma, moderately differentiated, consistent with colorectal origin. The resection margin is involved.
12. Liver, S6-3, hepatectomy --- Metastatic adenocarcinoma, moderately differentiated, consistent with colorectal origin. The resection margin is involved.
13. Liver, S6-4, hepatectomy --- Metastatic adenocarcinoma, moderately differentiated, consistent with colorectal origin. The resection margin is involved.
14. Liver, S6-5, hepatectomy --- Metastatic adenocarcinoma, moderately differentiated, consistent with colorectal origin. The resection margin is involved.
15. Liver, S4, hepatectomy --- Metastatic adenocarcinoma, moderately differentiated, consistent with colorectal origin. The resection margin is involved.
16. Liver, S7, hepatectomy --- Metastatic adenocarcinoma, moderately differentiated, consistent with colorectal origin. The resection margin is involved.
17. Liver and gallbladder, S4,5,8, hepatectomy --- a. Metastatic adenocarcinoma, moderately differentiated, consistent with colorectal origin. The resection margin is involved. b. Autolysis of gallbladder.

Ancillary study for diagnosis:
IHC stains for MLH1, MSH2, PMS2 and MSH6 are done on section Z. All above markers are preserved.

Prognostic and predictive factor:
1. Tumor size: 2.3 x 2 cm.
2. Depth of invasion: Pericolorectal tissue.
3. Histologic grade: G2: Moderately differentiated.
4. Angiolymphatic invasion: Not identified.
5. Perineural invasion: Not identified.
6. Tumor deposits (discontinuous extramural extension): Not identified.
7. Macroscopic tumor perforation: Not identified.
8. Surgical Margins: a) Proximal Margin: Uninvolved by invasive carcinoma, distance of tumor from margin: 6.5 cm. b) Distal Margin: Uninvolved by invasive carcinoma, distance of tumor from margin: 2 cm. c) Radial or Mesenteric Margin: Uninvolved by invasive carcinoma, distance of tumor from margin: 9.5 cm.
9. Lymph node status: Uninvolved (0/25).
10. Extranodal involvement: Not applicable.
11. Tumor regression grading S/P CCRT: No known presurgical therapy.
12. Type of polyp in which invasive carcinoma arose: Not applicable.
13. Additional pathologic findings: Not identified.
14. Pathological TNM Stage: pT3N0M1a(According to the eighth edition, American Joint Committee on Cancer Staging Guidelines for Tumors).
15. TNM descriptors: Not applicable.

Gross description:
The specimen consists of 1) a segment of colon, 10 cm in length and 4 cm in proximal cut end circumference, 5 cm in distal circumference, and mesocolic soft tissue. A gray tan firm annular elevated and centrally ulcerative tumor, 2.3x2 cm, is noted, 2 cm to the nearest cut end. On cross section, the tumor invades to the mesocolic soft tissue with 9.5 cm to the radial margin. The both cut ends are free of tumor grossly. 2) IMA root, a piece of tan yellow soft tissue, 2x1x0.4 cm. 3) distal cut end, a piece of tan mucosa-covered soft tissue, 2.5x2x1 cm. 4) "LL1", a piece of liver tissue, 1.5x1x0.6 cm. 5) "LL2", a piece of liver tissue, 2x1.5x1 cm. 6) "LL3", a piece of liver tissue, 2x1.2x1 cm. 7) "LL4", a piece of liver tissue, 3x3x1 cm. A gray ill-defined tumor, 2.8x0.8x0.8 cm is noted in it, with resection margin involvement. 8) "LL5", 2 pieces of liver tissue, up to 1.2x0.4x0.4 cm. 9) "LL6", a piece of liver tissue, 2.2x1.5x1.8 cm. 10) "S6-1", a piece of liver tissue, 2.6x1.7x0.6 cm. 11) "S6-2", a piece of liver tissue, 2x2x0.4 cm. 12) "S6-3", a piece of liver tissue, 2x1.5x1 cm. 13) "S6-4", a piece of liver tissue, 3x2.6x2 cm. 14) "S6-5", a piece of liver tissue, 2.5x1x0.4 cm. 15) "S4", a piece of liver tissue, 3.2x1.5x1 cm. 16) "S7", a piece of liver tissue, 3.7x3x0.7 cm. 17) "S4,5,8+GB", a piece of liver tissue, 370 gm and 13.5x12x4.2 cm, with gallbladder, 5.8x3 cm and 0.1 cm in width. On cut, some gray fragile tumors, up to 3.5x3x3 cm are found, and they are very close to the resection margin. All for section: A) specimen 2 C) specimen 4 D) specimen 5 E) specimen 6 H) specimen 8 L) specimen 11 Q) specimen 14; Representative part for section: B) specimen 3 F-G) specimen 7 J) specimen 9 K) specimen 10 M) specimen 12 N-P) specimen 13 R) specimen 15 S) specimen 16 T-V) specimen 17 W) gallbladder X) proximal cut end Y) radial cut end Z-AC) colon tumor AD-AG) LNs.

Note:
1. The interpretation of MMR panel and recommended studies are as below: (1) No loss of nuclear expression of MMR proteins: low

- probability of microsatellite instability-high (MSI-H) . (2) Loss of nuclear expression of MLH1 and PMS2: testing for methylation of the MLH1 promoter and/or mutation of BRAF is indicated (the presence of a BRAF V600E mutation and/or MLH1 methylation suggests that the tumor is sporadic and germline evaluation is probably not indicated; absence of both MLH1 methylation and of BRAF V600E mutation suggests the possibility of Lynch syndrome, and sequencing and/or large deletion/duplication testing of germline MLH1 may be indicated). (3) Loss of nuclear expression of MSH2 and MSH6: high probability of Lynch syndrome (sequencing and/or large deletion/duplication testing of germline MSH2 may be indicated, and, if negative, sequencing and/or large deletion/duplication testing of germline MSH6 may be indicated). (4) Loss of nuclear expression of MSH6 only: high probability of Lynch syndrome (sequencing and/or large deletion/duplication testing of germline MSH6 may be indicated). (5) Loss of nuclear expression of PMS2 only: high probability of Lynch syndrome (sequencing and/or large deletion/duplication testing of germline PMS2 may be indicated). (6) Please note: There are exceptions to the above IHC interpretations. These results should not be considered in isolation, and clinical correlation with genetic counseling is recommended to assess the need for germline testing.
  2. This case has been peer reviewed by two doctors.
  #T-59472_2 #M-81403_2 2 1105 S0000F
  #T-C4560_2 #M-09183_2 0 1105 000000
  #T-62000_2 #M-81406_2 2 1100 000000

其它

出院時情況

- 3.改門診治療

出院指示

藥名	劑量	劑量單位	用法	頻次	數量	數量單位	現況	天數	單位
Megest susp 120mL	10	ML	PO	QD	1	ML	TH	5	天
Herbesser tab 30mg	1	TAB	PO	TIDAC	15	TAB	TH	5	天
(口)Takepron OD tab 30mg	1	TAB	PO	QDAC	5	TAB	TH	5	天
Ultracet tab	1	TAB	PO	Q6H	20	TAB	TH	5	天
Amtrel tab 5/10mg	1	TAB	PO	QD	5	TAB	TH	5	天
Tresiba FlexTouch 300U/3mL	15	U	SC	QD	1	U	TH	5	天
MOSAD tab 5mg	1	TAB	PO	TID	15	TAB	TH	5	天
Flexpen Novorapid	8	U	SC	TIDAC	1	U	TH	5	天
110mg Pradaxa cap	1	CAP	PO	QDPC	5	CAP	TH	5	天
(口)Rosis tab 40mg	1	TAB	PO	QD	5	TAB	TH	5	天
Kombiglyze XR tab 5/1000	1	TAB	PO	QD	5	TAB	TH	5	天

出院計劃

主治醫師 2062F 蔣鋒帆 / 住院醫師 2301H 林陳彥

正式摘要(電子簽章：000000190725757)

編輯 W85 / W85 列印 個人範本 離開

doctorRank:B
